# Supplementary material for: Physiology and Transcriptomics Reveal Divergent Strategies of Mycorrhiza‐Mediated Drought Adaptation in Poplar
Source: Plant Cell Environ. 2026 Apr 6;49(8):5078–94. doi: 10.1111/pce.70511 (PMC13353687; doi:10.1111/pce.70511)
Supplement: Supplementary file 1 — Supplementary Figure S1: Relationship between poplar N content and biomass. Supplementary Figure S2: Number of leaves shed by poplars during a 4‐week experimental time. Supplementary Figure S3: Network analysis of DEGs in the GO term “Protein Folding”. [file PCE-49-5078-s001.pdf]

Supplementary figures

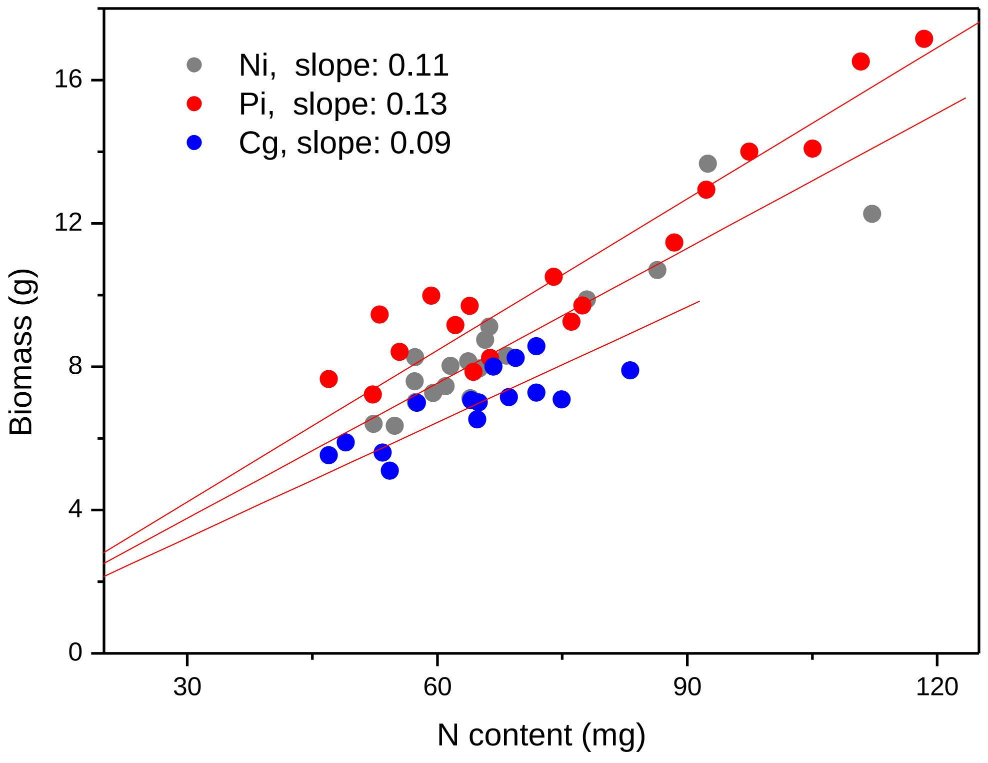

Supplementary Figure S1: Relationship between poplar N content and biomass. Linear regression models were applied to poplars colonized with *P. involutus* (Pi), *Cenococcum geophilum* (Cg) and non-inoculated (Ni) poplars. Each point represents an individual plant. R^2adj > 0.9, $p < 0.001$ for all variants.

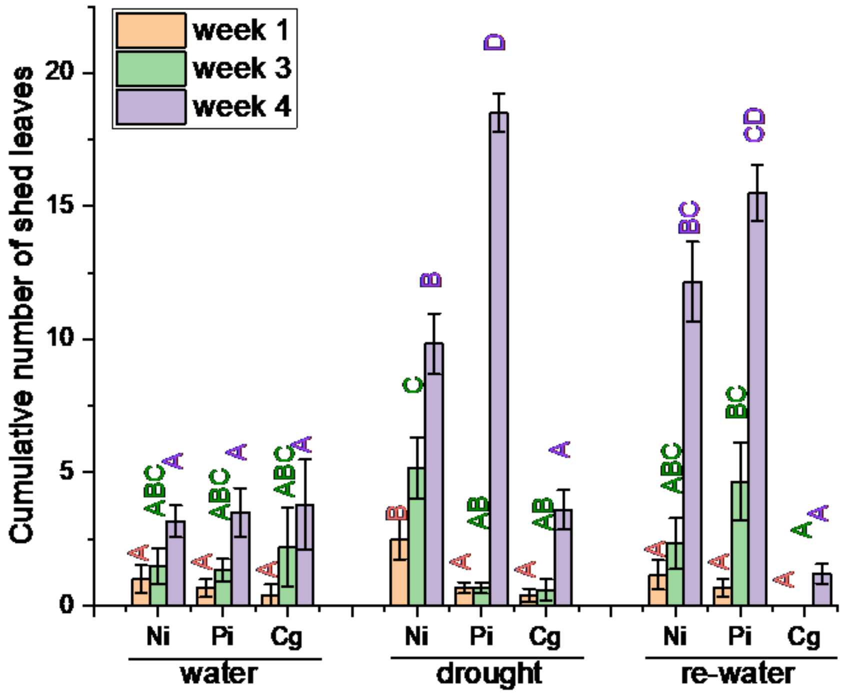

Supplementary Figure S2: Number of leaves shed by poplars during a 4-week experimental time. The bars indicate the cumulative leaf loss of well-watered, drought-stressed and rewatered plants, which were either non-inoculated (Ni) or colonized with Pi: *Paxillus involutus* or Cg: *Cenococcum geophilum* Fr.. Bars indicate means ± SE (n = 5 to 6 plant per treatment). Significant differences at $p \leq 0.05$ are indicated by different letters (Two-way ANOVA and post-hoc Tukey HSD test). Different letters indicate differences across treatments (orange: 1st week, green: 3rd week, purple: 4th week).

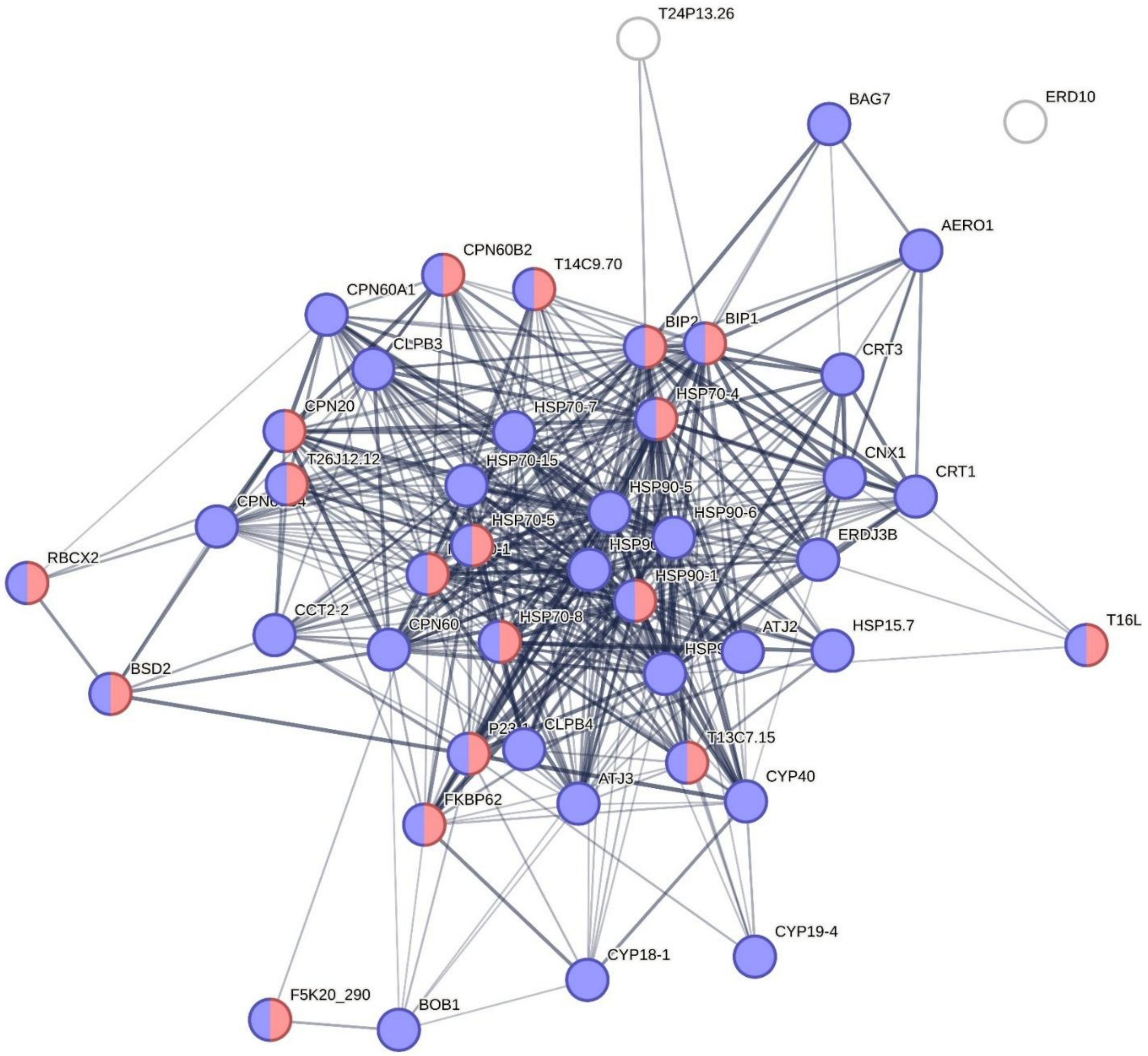

Supplementary Figure S3: Network analysis of DEGs in the GO term „Protein Folding". Blue: protein folding, Red: chaperone mediated protein folding. A total of 45 genes were identified that were significantly affected. Number of edges: 429, average node degree: 19.1, avg. local clustering coefficient: 0.703, expected number of edges: 25, PPI enrichment p-value: < 1.0e-16. Net works generated by *String* (https://string-db.org/) provided by Szklarczyk D, Kirsch R, Koutrouli M, Nastou K, Mehryary F, Hachilif R, Annika GL, Fang T, Doncheva NT, Pyysalo S, Bork P, Jensen LJ, von Mering C (2023). The STRING database in 2023: protein–protein association networks and functional enrichment analyses for any sequenced genome of interest. Nucleic Acids Res. 6:D638-646.
